# Australian integrative oncology services: a mixed-method study exploring the views of cancer survivors

**DOI:** 10.1186/s12906-018-2209-6

**Published:** 2018-05-09

**Authors:** Jennifer Hunter, Jane Ussher, Chloe Parton, Andrew Kellett, Caroline Smith, Geoff Delaney, Eleanor Oyston

**Affiliations:** 1 NICM Health Research Institute, Western Sydney University, Campbelltown Campus, Locked Bag 1797, Penrith, NSW 2751 Australia; 2 Menzies Centre for Health Policy, Sydney School of Public Health, Faculty of Medicine and Health, The University of Sydney, Camperdown, NSW Australia; 3 Translational Health Research Institute, Western Sydney University, Penrith, NSW Australia; 4 South-Western Sydney Clinical School, Faculty of Medicine, University of New South Wales, Sydney, NSW Australia; 5 Cancer Services, South Western Sydney Local Health District, Warwick Farm, NSW Australia; 6 Ingham Institute of Applied Medical Research, Liverpool, NSW Australia; 7 Oncology Massage Limited, Deakin, Geelong, ACT Australia; 8 Oncology Massage Global, Googong, NSW Australia

**Keywords:** Cancer survivors, Complementary medicine, Culturally and linguistically diverse background, Health service research, Integrative medicine, Integrative oncology, Mixed method

## Abstract

**Background:**

The significant use of traditional and complementary medicine (T&CM) by cancer survivors is well documented. The aim of this study was to explore cancer survivors’ views on integrating T&CM services with conventional cancer care.

**Method:**

A mixed-method study design with an emphasis on qualitative methodology was used to conduct and analyse four focus group interviews and an on-line survey. Purposive sampling recruited 33 cancer survivors and caregivers from Arabic, Vietnamese, Chinese and Anglo-European Australian backgrounds who participated in one of four focus group interviews, and 121 cancer survivors who responded to an on-line survey. The inductive thematic analysis was augmented with a descriptive statistical analysis.

**Results:**

Most participants had used T&CM therapies or consulted T&CM practitioners as an adjuvant during and/or after their initial cancer treatment. Two themes emerged: ‘positive perceptions and experiences’ and ‘barriers and unmet needs’. Participants emphasised that T&CM was not a ‘luxury item’, rather it was considered important for managing side effects and comorbidities, rehabilitation and quality of life. A wide range of complex, interrelated barriers and solutions to IO service provision and access were identified. Structural barriers included inadequate service provision, medical practitioner attitudes, logistical constraints and funding. Personal barriers were influenced by the severity of impairment and disability; attitudes, beliefs and knowledge about T&CM; and available resources (e.g. finances, time, transport). Unmet need and inequitable access was exacerbated by geographical location, ethnicity and ability to pay. There was a mismatch between where participants were accessing T&CM services and their preference for IO service delivery. Participants perceived hospital-based IO services availability to have several benefits, including the T&CM practitioners having more expert knowledge about cancer care, the convenience of co-locating oncology services, and potentially lower out-of-pocket costs.

**Conclusion:**

Patients’ use, preferences and needs for T&CM services in the oncology setting are important for informing service provision. Inequitable, unmet need reflected the increasing demand and expectation from patients for their oncology teams to be well informed about the benefits, risks and indications for T&CM use, and for the public and private health sectors to formally integrate and fund IO services.

**Electronic supplementary material:**

The online version of this article (10.1186/s12906-018-2209-6) contains supplementary material, which is available to authorized users.

## Background

The significant use of traditional and complementary medicine (T&CM) by cancer survivors is well documented [1–4]. Cancer survivors use T&CM for a variety of reasons that include managing treatment-related side effects and symptoms of cancers, enhancing the effectiveness of cancer treatment, prolonging life, improving quality of life and affirming self-efficacy [5, 6]. Among cancer survivors who do not use T&CM, barriers such as the cost and lack of time, fear and distrust, and lack of evidence are cited as reasons [7].

The term integrative oncology (IO) refers to the combining of T&CM interventions or services with conventional cancer care [8]. In this context, T&CM is mostly used as an adjuvant, rather than an alternative to standard cancer treatment [9]. In response to the significant use and demand for T&CM by cancer survivors, IO aligns with the paradigm shift towards active participation where patients are no longer encouraged to be passive recipients of cancer care [10]. Cancer survivors have expressed a preference to involve their medical team in decisions about T&CM use with and for oncology services to provide T&CM services [11]. IO acknowledges the increasing role that cancer survivors have in managing their own care and the need to build therapeutic alliances that respect the preferences and values of cancer survivors [9]. Similar to ‘integrated care’, IO aims to reduce the fragmentation of oncology services [9, 12].

Although interest in the implementation of IO services in Australia and abroad continues to grow, questions remain about appropriate service development [13–18]. As for any quality improvement process, understanding patient needs and experiences are essential [19, 20]. Research however has mostly focused on the prevalence of T&CM use, motivators and barriers for use, patient perceived outcomes, and challenges with patient-clinician communication. Less is known about cancer survivors’ experiences with IO services and their views on what the integration of conventional cancer services with T&CM might look like, the types of services they need, and preferences for service delivery. If T&CM is to be successfully integrated with conventional cancer care services further research that includes the views of diverse cancer patient population groups is required.

The estimated period prevalence of T&CM use by cancer survivors in Australia is 43% (95% CI: 19–67%), and like other comparable countries in Europe and Northern America, these rates have dramatically increased over the past 40 years [3]. Despite the high rate of use and demand, IO service provision in Australia lags with less than a quarter of cancers services in the public and private health sectors providing any type of IO or supportive care [21, 22]. Access to IO is further limited by affordability due to substantial out-of-pocket costs for cancer care that is not provided by publicly funded hospitals [23]. Public health insurance rebates are only available for integrative medicine services and medical acupuncture provided by medical practitioners; and only some of the private health insurance schemes offer partial rebates for services provided by T&CM practitioners.

It is important to provide a voice for the culturally and linguistically diverse (CALD) communities utilising cancer services. Evidence suggests that survival rates for many CALD cancer survivors are lower than those of Anglo-Australian background [24]. English ability and knowledge of the health system are important contributors, and have been found to be the strongest predictors of poor psychological and quality-of-life cancer outcomes [25]. After controlling for demographic and disease, CALD cancer survivors from any ethnic background have up to four times higher risks of depression and anxiety compared with their Australian-born Anglo counterparts [25]. They also experience higher unmet needs for information and help with physical problems that persist several years after initial cancer diagnosis [24].

There is evidence of unmet IO healthcare needs for cancer survivors from CALD backgrounds. In a series of focus group interviews with cancer survivors of Greek, Cantonese, or Mandarin speaking survivors who were living in Australia, although questions about T&CM were not asked by the researchers, participants still expressed unmet IO needs, such as difficulties with finding a traditional medicine doctor and accessing reliable expert information to help them manage their cancer and side effects [26]. Indeed, there is good evidence that a range of T&CM interventions can improve mental health and wellbeing [27], suggesting a potentially important role for IO in providing both culturally appropriate and clinically effective services.

In light of the growing use and demand for IO services in Australia, this study aimed to explore cancer survivors’ views and experiences with IO, service needs, preferences, enablers and barriers; and to ensure the views of some of the culturally and linguistically diverse (CALD) population groups that are underrepresented in Australian cancer research were included.

## Methods

### Design

A mixed-method study design with an emphasis on qualitative methodology was used to conduct and analyse four focus group interviews and an on-line survey.

### Sample and recruitment

Purposive sampling was used to recruit cancer survivors from Anglo-European, Arabic, Chinese and Vietnamese Australian backgrounds living in Sydney for four focus group interviews and an on-line survey. The rational for selecting the three CALD groups reflected the prevalence of these communities in South West Sydney region where the research was located. Although Australian and Torres Strait Islander peoples are also common in this region, these communities were not included due to the logistical constraints of the project.

Focus group participants were recruited through the distribution of information sheets in English, Arabic, Chinese, and Vietnamese by collaborating hospital clinics, culturally specific cancer support services and a hospital-based cancer wellness centre. Interviewees were offered AUD25 gift card as a reimbursement for expenses. Survey participants were recruited through Facebook networks such as the National Breast Cancer Foundation, Oncology Massage Ltd., and natural therapies organisations. Recruitment strategies for the survey anticipated mostly sampling cancer survivors from Anglo-European Australian backgrounds and T&CM users.

### Procedure and instruments

The focus groups were designed to facilitate the in-depth examination of subjective experiences, meaning, and perceived consequences of T&CM use and its integration in the context of cancer services. Additional file 1 outlines the interview schedule. Basic demographics were collected via a simple paper questionnaire that was written in English. The interviewers and translators assisted participants when language was a barrier. Two experienced interviewers (author CP and a graduate assistant AH, see acknowledgements) used an “extended conversation” technique ([28], p. 96), in which the wording and formatting of questions was flexible to suit the particular context and experience of the participants. There were no prior relationships established between researchers and participants prior to study commencement. The interviews were conducted in hospital and community locations.

Interpreters were used for the three CALD groups. The interpreter for the Chinese group was accredited in Mandarin and Cantonese. Interpreters were asked to translate the actual wording of each participant’s talk rather than summarising responses to capture the detail of participant accounts. A questionnaire was used to collect anonymous information about the demographic and cancer history of participants. The interviews ran for approximately 60 min and were audio-recorded and transcribed verbatim, with the resulting transcripts then read in conjunction with the audio recording to verify any errors in transcription.

The online survey was administered through SurveyMonkey [29]. It was anonymous and consisted of 26 closed and open-ended questions examining participants’ views on T&CM in the context of cancer (Additional file 2). This included demographic and cancer history questions, whether participants had used T&CM services and therapies, the context of their use, where they would like to have received T&CM, and enablers and barriers using T&CM as part of their cancer care. Open-ended questions were used to collect the demographic information; eleven of the close-ended questions were followed by an open-ended question asking for an explanation to the response; seven multiple choice questions included an open-ended option “Other (please specify)”; and the survey ended with a section for further comments. The questionnaire was designed to collect data compatible with the topics discussed in the interviews.

Although prayer and spiritual practices are one of the most commonly used non-biologically based CM therapies both in Australia and abroad [3, 30, 31], questions about prayer were excluded. The focus of the research was IO services and in Australia, pastoral care services are not classified as an IO service [3, 30, 31]. The researchers recognise that the boundaries can be blurred, as T&CM wellbeing services such as meditation and mindfulness may draw on religious philosophies and traditions, and spirituality (rather than religiousness) is considered to be a domain of ‘holistic health’ [32, 33]. Nevertheless, the decision was made that prayer and pastoral care were outside the scope of this research project.

### Analysis

Thematic analysis of the of the interview transcripts and open-ended survey responses was conducted [34]. The style of analysis adopted was inductive with the development of themes being data driven. A subset of the interviews was independently read by two of the authors to identify first order codes, such as benefits, negative experiences, practitioner attitudes and awareness. The entire data set (interview transcripts and survey answers) was then coded using NVivo Version 11 [35]. The coded data was independently reviewed by two of the authors. Codes were then grouped into higher order themes. A careful and recursive decision-making process which involved checking for emerging patterns, for variability and consistency, and making judgements about which codes were similar and dissimilar was used to develop a thematic map of the data outlining core themes and subthemes.

Quantitative data from the survey was analysed using descriptive statistics in IBM SPSS Statistics for Windows, Version 24.0 [36]. Some of the open-ended responses to survey questions were also recoded into categorical groups. Recruitment for the survey was designed to augment the qualitative results from the interviews rather than create a representative, generalisable sample. When calculating the percentage of responses to a question, missing data was therefore excluded from the denominators.

To minimise bias regarding the coding and interpretation of the qualitative data, the research team undertaking the analysis was comprised of members with and without a background in IO or T&CM. Five of the researchers were female and three were male.

## Results

### Sample and response rates

Purposive sampling recruited 33 cancer survivors and caregivers from Arabic (*n* = 11), Vietnamese (*n* = 9), Chinese (*n* = 7) and Anglo-European Australian (*n* = 6) backgrounds for the focus group interviews (Table 1). Four of the Arabic focus group participants were caregivers. Although their recruitment was unintended, the participants’ caregivers were nevertheless invited to participate in the focus group discussion.

**Table 1:** Characteristics of focus group participants

Participants	*n* = 33	%
Cancer survivors	29	88
Caregivers (Arabic)	4	12
Average age (years)	*n* = 31	SD
Cancer survivors & caregivers^a^	64.0	9
Gender	*n* = 33	%
Female	23	70
Male	10	30
Ethnicity	*n* = 33	%
Arabic	11	33
Vietnamese	9	27
Chinese	7	21
Anglo-European	6	18
Median years since diagnosis	*n* = 29	range
Cancer survivors	6	0.3–30

^a^data missing for 2 caregivers

For the online survey, 121 cancer survivors responded (Table 2). Most of the survey questions were not compulsory; as such, response rates to questions ranged from 100% (*n* = 121) down to 67% (*n* = 84). Mostly, this was due to skipped questions throughout the survey with only 14 (12%) not completing the survey.

**Table 2:** Characteristics of survey respondents

Average age (years)	*n* = 121	SD
	59.6	11.1
Country of Birth	*n* = 121	%
Australia (2 ATSI^a^)	92	76.0
New Zealand (2 Maori, 2 Samoan)	6	5.0
Europe / United Kingdom	17	14.0
Africa	3	2.5
Asia	3	2.5
Languages spoken at home	*n* = 120	%
English	116	96.7
English and another language	4	3.3
Median years since diagnosis	*n* = 121	range
	3.6	0.3–36.5
Diagnosis	*n* = 121	%
Breast or gynaecological cancer	82	67.8
Haematological, bowel, skin or lung cancer	25	20.7
Prostate cancer	4	3.3
Other (includes multiple primary cancers)	10	8.3
Current treatment	*n* = 121	%
No treatment	65	53.7
Long-term treatment / rehabilitation	46	38.0
Active treatment	10	8.3
Treatment in last 12 months^b^	*n* = 120	%
None	50	41.6
Surgery	38	31.6
Radiotherapy	31	25.8
Chemotherapy	26	21.6
Other cancer medication	25	20.8

^a^ATSI - identified as Aboriginal and Torres Strait Islander

^b^more than one response permitted

### Participant characteristics

The average age of participants was 64 and 60 years for the focus group and survey respondents respectively; and the median years since cancer diagnosis was 6 years (range: 0.3–30 years) and 3.6 years (range: 0.3–36.5 years). The majority of focus group participants were female (Table 1). The survey accidentally omitted a question on gender. Cancer diagnosis, however, demonstrates that at least 82 (68%) respondents were female (Table 2). Focus group participants were not asked about their cancer diagnosis.

The survey sample of 121 respondents was biased towards English speaking Anglo-European Australians who were females, with a diagnosis of breast cancer, and oncology massage users. Just under half (46%) of the respondents were currently engaged in either active treatment (e.g. surgery, chemotherapy or radiotherapy) or long-term treatment (e.g. hormone suppression medication, other medication, or rehabilitation), and 59% had received some form of treatment for their cancer in the previous 12 months.

All participants in the Arabic focus group categorically denied having any experience with any type of T&CM as part of their cancer care. Participants in the other three focus groups had used T&CM during or after their initial cancer treatment. Most of the survey respondents (92.2%, *n* = 107/119) had used a range of T&CM modalities and/or products such as vitamins, minerals, and herbs as part of the cancer care (Table 3). No-one had used T&CM instead of, nor as an alternate, to their standard cancer treatment.

**Table 3:** Survey participants’ use of traditional and complementary medicine

T&CM use since cancer diagnosis	*n* = 116	%
Both T&CM modalities & products	74	63.8
Only T&CM modalities	28	24.1
Only T&CM products	5	4.3
Any T&CM use	107	92.2
No T&CM use	9	7.8
T&CM modalities commonly used since cancer diagnosis^a^	*n* = 116	%
Massage or touch therapy	93	76.9
Mental wellbeing (e.g. meditation, art/music therapy)	61	50.4
Movement modalities (e.g. yoga, tai chi, massage)	41	33.9
Naturopathy or nutritional medicine	38	31.4
Acupuncture or Chinese medicine	17	14.0
Body Alignment (e.g. chiropractic, osteopathy, cranio-sacral)	15	12.4
Indigenous Australian healing practices	1	0.8
Other	2	1.7
Timing of T&CM use^a^	*n* = 96	%
Before commencing cancer treatment	14	14.6
During cancer treatment	44	45.8
Following cancer treatment	75	78.1

^a^more than one response permitted

### Thematic results

In the final stage of mixed method analysis, two overarching themes emerged. The first theme, “positive perceptions and experiences”, predominantly drew on the data from the focus group interviews. Seven subthemes were identified: 1) perceived positive impact on cancer survival, 2) perceived positive impact on side-effects and recovery, 3) perceived positive impact on co-morbidities, 4) perceived positive impact on wellbeing, 5) downplaying negative outcomes, 6) positive experiences with T&CM practitioners, and 7) positive experiences with IO services.

The second theme, “barriers and unmet needs”, drew on data from both the survey and the focus group interviews. Five subthemes were identified: 1) lack of availability of IO services, 2) difficulties with referral pathways and information, 3) absence of medical practitioner support, 4) difficulties with access, and 5) cost of care. Two other interacting subthemes were identified a) structural barriers and b) personal barriers that applied to the five other sub-themes. For example, difficulties with accessing IO services reflected both structural barriers, such as only hospital-based services were offered, and personal barriers, such as being too unwell to travel to the hospital to access the service.

In the presentation of the results below, the survey data reports the frequency of responses as they relate to the core themes and subthemes that emerged from the qualitative analysis. The meaning and consequences of these themes draws on the qualitative analysis of the interviews and the open-ended survey items. Details of the data source – focus group (FG) or survey – are only provided for longer quotes that are presented in Tables 4 and 5.

**Table 4:** THEME 1 - Positive perceptions & experiences

Perceived positive impact on cancer survival	
• Interpreter: *“The cancer in my lungs had developed and went up to the brain. Terminal, it was just terminal. So, in 2000 I took Chinese medicine for one year and it stopped”*. (Vietnamese FG)	
• *“The final result from this* [food, exercise and Chinese medicine] *is your immune system becomes strong, balanced. Then you will get healthy and you have a chance that it will fight each other – the good guy fighting the bad guy.”* (Chinese FG) • *“It is very helpful to me and many of the people who also practise qi gong were diagnosed five or ten years ago and they are still living.”* (Chinese FG)	
Perceived positive impact on side-effects and recovery	
• Interpreter*: “It’s just after drinking the Chinese medicine, it really helped me with eating because after taking chemo, I was unable to eat… Yeah. I’ve been drinking Chinese medicine now for over a year”* (Vietnamese FG) • *“So, the doctor said “You can have additional therapy like radiotherapy, but – or you can wait…” So, I was thinking, well, what can do in meantime? I know I have the cancer, but they are minor. So, then I think about other complementary therapy like a supplement, like what* [another participant] *told and I did went to see a traditional herbal Chinese doctor… after recovery from the surgery, I’m back to normal.”* (Chinese FG) • Interpreter: *“Since I started meditation, I’ve reduced on the painkillers.”* (Vietnamese FG)	
Perceived positive impact on co-morbidities	
• *“… But I found with the acupuncture and the massage in particular, apart from the side effects that you get from the traditional treatments – your chemotherapy, your radiation …. if you had any other ailments prior to cancer, they continue to exist throughout the whole cancer … journey. And those supplementary therapies help with that as well as the other, because when you have got cancer, those other things suddenly seemed magnified.”* (Anglo-European FG) • *“The massage is great for me because I have all the underlying* [health problems] *are also a part of me… The doctor said, “We can’t give you chemo that these guys have had,” …he said to me, “If we give you normal chemo, your lungs will pack in before you do.” Now, he said to me* [there was a change in the treatment plan] *and I said, “Why?” He said, “because you’re not as sick as you should be” and I said, “Well, is that a positive?” …and I just feel the complementary things that they do give us, the massage, to me, is a Godsend.”* (Anglo-European FG)	
Perceived positive impact on wellbeing	
• *“I haven’t tried anything else but the massage. I found it really relaxing especially the last time I went. That’s about a couple of weeks ago. I have a problem. I was tired. I wasn’t having any energy. I was really depressed and when she took me in for about 45 min, when I come out and I felt like, “God bless you. You’re here to help people.” It was so beautiful, and so relaxing, so good. So you come out and you think, ‘God, please help those ladies and help us to help others.’”* (Anglo-European FG) • *“Yeah, so I’ve – started the supplement. I find I’m full of energy. I feel well again. I feel great. So therefore, I continued taking the supplement so hopefully that it will bring a good immune system to me that I can continue to work and do my normal stuff.”* (Chinese FG) • *“There’s the physical symptoms and so on that you get, and then there’s the emotional. Even though they may not be necessarily a psychologist or whatever, I have found that it does help you with the emotional and mental side of it as well* [as] *the whole thing.”* (Anglo-European FG)	
Downplaying negative outcomes	
• *“And then just by experience because somebody take, very good – oh, this medicine is very good. And everybody take. Somebody take, very good really, and somebody die. But they die, they kept very quiet. They never say die because of this medicine. But if somebody become good, they say, “I take this medicine. So, oh, good.” And then there’s – of course they will promote. They always tell a good story.”* (Chinese FG)	
Positive experiences with T&CM practitioners	
• *“I can go to my local guy and get a massage and he’ll get sort of right into the deep tissue and all the rest, but it’s not the same. I don’t come out of there feeling the same as I do when I leave here, so it’s not just getting a massage or just getting acupuncture… It’s a different feel..”,* • *“It’s that person that is dealing with cancer patients… they have that specific training with oncology. I mean they’re not oncologists obviously, but they have some training and awareness of – of the whole thing”* (Anglo-European FG) • *“See, that sort of stuff that you would – you probably could sit down and have a really good discussion with a naturopath… Yeah… Because a lot of people – in my opinion and just my opinion, have no training whatsoever, but a lot of people will take vitamin supplements and so on. If you’re taking vitamin D, and you don’t need it, it’s wasted.”* (Anglo-European FG)	
Positive experiences with IO services	
• *“I think to have it close by to where the other treatments are taking place helps, because I was able to go from radiation and then after I finished my treatment, then come up to the wellness centre and continue with whatever it was that I needed.”* (Anglo-European FG)

**Table 5:** THEME 2 - Barriers and unmet needs

Lack of availability of IO services	
• *“It’s not fair to come here only once a fortnight or once in 20 days because you don’t get the benefit* [of acupuncture] *… The massage therapist, half an hour is not enough. You have people with limited mobility and the time that is taking to get into the room, to get undressed, you’ve got a questionnaire to fill out before the treatment and then after the treatment … there is a limit on the number of times I can access oncology massage following my cancer treatment.”* (Anglo-European FG) • *“I think it should be better for each hospital have one [T&CM] department. You know why? Some time we went to the* [hospital] *– they got more Chinese patients. They know more knowledge about the Chinese medicine. Even we went to the* [hospital] *– they don’t know anything. They only have Western people.”* (Chinese FG)	
Difficulties with referral pathways and information	
• *“We don’t know anything about it. Nobody tell us – no information about that and how. There’s a lack of information.”* (Arabic FG) • *“We need to know what the herbs* [are that can] *help us – what sort of herbs – millions of herbs. What sort of herbs, what sorts of food is good?”* (Arabic FG) • *“I’ve been through this journey for a long time and I’m still suffering, but when I go to the doctor, the doctor just tell me to take this tablet before food, this tablet after food, and this. He never mentioned any other support or any other alternative that we can do”.* (Arabic FG) • *“But only a couple of months ago,* [the doctor] *goes … “Why don’t you try acupuncture?” And I thought, “Oh, great. Okay, something new.” But why wasn’t it mentioned one and a half years ago? … why did I have to get to breaking point to be told – look, what’s available?”* (Anglo-European FG) • “Doctors should be encouraged to use [T&CM] therapies for their patients in conjunction with their treatment not instead of, or as an after-thought.” (Survey) • *“The best* [source for information and referrals] *is GP, because you always go to your GP, and he has to know. He has to have the education to know all that, to tell us what to do, and if you go to the hospital, you have a long waiting time…* [yet] t*he GP doesn’t have enough information. The GP said, “Just do whatever they* [the oncologist] *tell you…” but the oncology told us about the treatment, that’s it! Maybe you can know the information from the nurse or from social worker?”* (Arabic FG) • Interpreter: *“There’s many channels that you can have access to information related to complementary medicine for cancer patients. We can hear about from your family members, from community, from GPs...* [the problem is that] *we receive very conflicting information and it’s not well-organised.”* (Chinese FG) • *“The where is not as important as knowing what is available - being informed - preferably by not relying on one source to provide information.”* (Survey)	
Absence of medical practitioner support	
• *“My oncology doctor tell me, “I don’t want to know”*.” (Chinese FG) • *“Maybe we’re just waiting for chemo or anything, but actually in the meantime, we’re taking other supplements, this sort of thing.”* (Chinese FG) • *“They should be told because they need to know if it interferes with treatment.”* (Survey) • *“It shouldn’t be the secret that it often seems. Treatment should be a team approach including support and acceptance”.* (Survey) • *“If my oncology team aware of my complementary therapies and they have given good results, it could help others.”* (Survey) • *“My team know I do complementary therapies but we do not discuss this as they are not open to it. I have no issues with them knowing as I am proud that I am being active in seeking self-help.”* (Survey) • *“Unfortunately, I was advised that many oncologist frown on these therapies. Personally, I was lucky enough to not come across this attitude but was simply told while the benefits of such therapies hadn’t been proved they hadn’t been disproved either.”* (Survey) • “[Doctors] *will not recommend anything* [that] *is not proved. And it is their duty and they’re professional”.* (Chinese FG) • *“I think traditionally, there’s always been this thing between doctors and alternative therapy practitioners.”* (Anglo-European FG) • *“Those who do receive education here in the Western world, they don’t have any faith in traditional Chinese medicine.”* (Chinese FG) • *“It shouldn’t be looked at as an ‘us and them’ thing, the patient needs to get well asap and different remedies, just as different chemos, work on different patients.”* (Survey) • *“I would prefer these complementary things to become more mainstream, and to not have to justify them as if I am some kind of gullible idiot.”* (Survey)	
Difficulties with access	
• *“Lack of energy is a big factor, making more appointments when you already have a lot of appointments puts me off. Having a massage therapist come to the house would be great!*” (Survey) • *“There was an extended period of time when I was certainly too ill to access anything that was not absolutely necessary.”* (Survey) • *“I would like to see a natural therapist, but as it is in another suburb, I haven’t had the energy and keep putting it off. I may do it soon.”* (Survey) • *“I just don’t have the time at the moment, as I am in the middle of daily radiation and it takes me 1 1/2 h to the cancer centre and 1 1/2 h back. It’s the travelling that wears me out, not the treatment.”* (Survey) • *“Transport is a problem for me to try to get to her private practice. I can’t do that. So, she’s got to go without”.* (Arabic FG, caregiver) • Interpreter*: “He said if it can be near the station. I think a lot of transport, I think, is an issue, isn’t it?”* (Arabic FG) • “*I’m picked up at 6:30 in the morning. My appointment might not be till 10:00 because their drop off there is 8:00 … their last pick up is at a certain time … And you’re exhausted at the end of the day.”* (Anglo-European FG) • *“I would have used more yoga but it wasn’t available at a time that suited and local classes were too intense. I also couldn’t find a suitable meditation class so only managed to do this on a breast cancer retreat and then privately.”* (Anglo-European FG)	
Cost of care	
• Interpreter: *“So now it’s common understanding amongst the cancer patients that with complementary treatment that the cost associated with it is really a huge burden on us besides the disease, it’s not affordable – financial pressure. This is one of the barriers.”* (Chinese FG) • “*All of us, we are a pensioner... We couldn’t pay* [for T&CM]”. (Arabic FG) • *“They are very expensive as an ongoing treatment given all the out of pocket expenses associated with cancer surgery.”* (Survey) • *“Complementary therapies are seen as a luxury and often expensive especially when income capacity is reduced due to illness.”* (Survey) • Interpreter: “*Yeah. So, same thing, because cancer is a chronic disease, it takes a long time for you to recover, not only for – mentally and physically. So, they really need the financial support from the government.”* (Chinese FG) • *“I reckon the government should pay for it. Why shouldn’t they? I mean the rich get richer, the poor get poorer. I mean it’s lucky for some people who’ve got – like me, I’ve got my life and personal accident insurance, but who else would have that?”* (Anglo-English FG) • *“We pay $4000 for the health fund – we only get such items – only $200* [in total rebates for T&CM services]. *How can we afford it?”* (Chinese FG) • Interpreter: “*Funded by the government, that will be good, but then if not, then of course, it’s the children that help out parents. So if the children don’t give, then I just have to live with that.”* (Vietnamese FG) • *“I went back to China to bring capsules here ... it’s much cheaper there.” (Chinese FG)* • *“I’m just conscious of people thinking when you go for the massage that it’s just about spoiling yourself. It’s like going to the day spa. It’s not day spa type massage. It’s therapeutic remedial massage.”* (Anglo-English FG) • *“So, that might be reason why there’s difficulty perhaps in getting continued funding and so on because it’s easy for people to think it’s just a – this is just a feel-good stuff. It’s not. And the same with the acupuncture, it’s not just feel-good stuff and I can’t emphasise that enough. It’s much, much more.”* (Anglo-English FG) • *“I myself struggle to pay for them*… *and they aren’t a luxury item they are a necessity to minimize damage and should be part of the Medicare rebate.”* (Survey)	

### THEME 1: Positive perceptions & experiences

Throughout the focus group interviews, participants reported a range of positive experiences and perceived benefits of using T&CM throughout their cancer journey (Table 4). One was of the belief that it could have a positive impact on cancer survival. Many discussed the positive impact of T&CM on side effects and recovery. T&CM was used more broadly for comorbidities other than just cancer care. Participants reported positive experiences with T&CM practitioners, emphasised their appreciation of professional T&CM care and identified benefits of an IO approach to service provision. Although the survey did not specifically ask about perceived benefits from T&CM use, the majority of survey respondents (95%, *n* = 92/97) stated that they would recommend T&CM to others, and 92% (*n* = 98/107) would consider using T&CM again.

### Perceived positive impact on cancer survival

One of the perceived benefits of T&CM that was identified during the focus group discussions was a positive impact on cancer survival, *“so the tumour – it remains the same. It didn’t develop more… Over time, I see my health start to get better”*. Another participant provided an account of the benefits of eating *“normal food, balanced food, good exercise”* combined with Chinese medicine for *“fighting”* cancer (Table 4).

### Perceived positive impact on side-effects and recovery

Participants gave accounts of using T&CM to *“enhance treatment and protect* [the] *body against side effects of treatment”* or *“to improve my general health”* after treatment (Table 4). This included the benefits of massage *“to improve my overall general health and to assist with managing lymphoedema”* and *“to assist my rehabilitation”;* vitamins *“to assist with side effects of chemotherapy induced menopause*”; meditation to *“reduce the painkillers”;* acupuncture to deal with *“pain on my legs”*; and tai chi and yoga *“to make you feel a little bit more important and a little bit more active”.*

### Perceived positive impact on co-morbidities

T&CM was also used to deal with concurrent health problems that exist alongside cancer-related symptomatology (Table 4). In supporting general health and comorbidities, the use of T&CM was seen as an important adjuvant to conventional cancer care by helping to improve tolerability and cancer outcomes.

### Perceived positive impact on wellbeing

Participants emphasised the important “*remedial”* and *“therapeutic”* role T&CM played in as an uplifting, healing experience that supports wellbeing amidst the stressors of cancer treatment (Table 4). The holistic approach of T&CM therapists and their ability to help with physical and emotional symptoms was also identified.

### Downplaying negative outcomes

Not all outcomes from T&CM use were positive, however, and another participant described a perception of a social bias regarding T&CM outcomes within his community (Table 4). Successes of T&CM were acclaimed, and failures suppressed. For others, despite uncertainty about any benefits, they continued to use T&CM.

### Positive experiences with T&CM practitioners

Participants reported positive experiences with T&CM practitioners and emphasised that practitioners with appropriate training helped ensure quality care (Table 4). Naturopaths were identified as having the necessary expertise for an in-depth discussion about supplement use. Acupuncture practitioners were described as *“so calm”* and as having *“that specific training with oncology”*, which meant that “*they have training and awareness of the whole thing”* resulting in reports of *“confidence”* in the outcomes of T&CM. Others noted a difference in quality between massage provided by the trained oncology massage therapists available at the hospital and general massage services provided in the community *“the massages outside aren’t any good”.*

### Positive experiences with IO services

Participants perceived hospital-based T&CM services to have several benefits, including the provision of a *“completely holistic sort of treatment plan”,* practitioners having expert knowledge about cancer care, the benefits of locating services close to the site of other cancer treatment, and lower out of pocket costs (Table 4). IO services were also provided through the participants primary care services, such as a General Practitioner (GP) who was also provided acupuncture services.

### THEME 2: Barriers and unmet needs

A lack of availability of IO services, difficulties with referral pathways and information, the absence of medical practitioner support, difficulties with access, and the cost of IO care were identified as the key barriers and unmet needs for IO services (Table 5). Only 15% (16/107) of the survey respondents reported that there were no barriers preventing them from using T&CM, or using more T&CM. Barriers could be further understood by considering the interrelationship between structural barriers and personal barriers. Examples of structural barriers included inadequate service provision, service location, medical practitioner attitudes, unclear referral pathways, lack of information, and lack of funding. Personal barriers for the individual and their caregivers were influenced by the severity of impairment and disability; attitudes, beliefs and knowledge about T&CM; and available personal resources (e.g. finance, time or transport).

### Lack of availability of IO services

Participants reported there were T&CM services that they would like to have used but could not. One of the reasons for this was inadequate service provision, including a lack of culturally appropriate T&CM services, and services in regional/remote areas (Table 5). Of the survey respondents, 36% (*n* = 39/107) reported that lack of availability of services either stopped them from using T&CM or from using more T&CM.

Both interviewees and survey respondents reported the absence of specific T&CM services or difficulties with finding T&CM practitioners knowledgeable in cancer care: *“I couldn’t find a decent massage therapist …who understood cancer, scars, and lack of skin sensation”*; *“reflexology wasn’t offered”*; *“it’s hard to find suitably trained providers”*. If T&CM services were available through the hospital, they were described as *“often very booked”*, with *“demand far, far greater than the service that’s available”* (Table 5). The time available for T&CM therapy sessions, when they were available, duration of treatment, and limits being placed on the total number of sessions, were also a focus of criticism. Several participants in the focus group discussions also reported a limited mindset and expertise on the part of hospitals in the provision of culturally appropriate T&CM services (Table 5).

### Difficulties with referral pathways and information

Even when hospitals were providing T&CM services, structural barriers, such as obtaining referrals and information about available T&CM services, remained an obstacle; however, “*once you come through that front door*, *you get that information. But it’s getting through that front door, somebody telling you that it exists”.* Inadequate information was given as the primary reason why no-one in the Arabic focus group had used T&CM nor IO services (Table 5).

Focus group participants reported frustration with their healthcare professionals not discussing T&CM options, being slow to refer, or receiving conflicting information about safety and indications, noting that the onus was on patients to question their healthcare professionals about T&CM options (Table 5). Often, it was up to the participant to seek out information or find a poster or leaflet in the oncology department advertising the IO, to which one participant remarked “*I didn’t see it”.* Other participants talked about being referred straight to conventional treatment, with a lack of consideration for T&CM adjuvant therapies (Table 5).

A disparity was found in the survey results between how participants wanted to receive referrals to T&CM and obtain information about T&CM and what they had received. When the survey participants were asked: “How would you like to be referred to a T&CM service”, 63% (*n* = 69/110) of respondents indicated they would prefer a referral via an oncologist or GP, with 22% (*n* = 25/110) indicating self-referral as the method of choice. Yet of the 94 survey respondents who reported they had accessed T&CM services, only 47% of respondents reported that a medical doctor (either a specialist or GP) had made the referral or recommendation and 46% had self-referred.

While T&CM users were finding ways to access T&CM in the community without a GP or oncologist, there was a clear preference for referrals to come from this quarter as medical practitioners were held in a position of expertise: *“I also take the doctors opinion ‘cause they have better knowledge”*. Other participants commented that GPs were better placed than specialists to distribute information and monitor T&CM use because they *“work with you like a family”* and patients had more frequent contact with GPs than their specialist medical practitioners (Table 5).

The need for trusted, reliable information from a variety of sources was emphasised throughout (Table 5). Participants gave accounts of wanting more information about T&CM from their GP – *“even a little that’s got one or two pages”, “there’s a lack of information” –* and for the provision of information and referrals by GPs be supported by other channels as well. Others preferred information sources and referrals were via social workers, pharmacists or community or religious leaders; books, magazines with diagrams, or succinct information presented in a pamphlet written by T&CM therapists; or *“through word of mouth from somebody who has been through the same or similar experience” or “friends”.* Although some focus group participants reported the use of websites, information from the internet was generally viewed as unreliable or difficult to access because *“nobody know how to use the internet”.*

### Absence of medical practitioner support

Participants identified ambivalent or negative attitudes from their healthcare professionals (particularly GPs and oncology specialists); difficulties with doctor-patient communication, including disclosure of use and building a therapeutic alliance; conflicting advice or a lack of knowledge about T&CM options; the necessity for scientific evidence; and professional and cultural divides (Table 5). In the survey, 13% (*n* = 11/84) of respondents selected “unsupportive healthcare practitioner / doctor” as a reason for not using T&CM or more T&CM.

Although many participants talked about their wish to freely discuss their T&CM use with their doctors, negative and ambivalent attitudes from the medical profession were reported to have an impact on patient disclosure of T&CM treatments (Table 5). These negative attitudes were at odds with the survey findings whereby virtually all respondents (98%, *n* = 109/111) opted for wanting their oncology team to know that they were using T&CM therapies and 63% (*n* = 69/110) preferred a referral from either their GP or oncologist.

Open discussion about T&CM use with doctors was seen as a way to improve *“coordinated care!”.* The *“oncology team and therapy teams should work together”* and “*be fully informed to reach the best outcome.”* Safety, interactions, contraindications and “*potential risks”* that T&CM may “*interfere with treatment”*. Disclosure of T&CM use to the oncology team was also described as a way to *“educate them about the benefits of using those therapies”* and prompt clinical inquiry, as the doctor *“can then ask how is it helping / why etc.”* This in turn may increase the likelihood of others benefiting from IO. For other participants, discussion about T&CM was seen as a way of reminding their oncology team about the importance of self-care and holistic healthcare (Table 5).

Not all participants however, wanted to disclose their T&CM use or thought it was appropriate. When one participant from the Vietnamese focus group was asked if they had talked to their doctors, or wanted to talk, about the Chinese herbal medicines they were taking, on both occasions the answer was *“no”,* as they perceived the role of their GP was only to provide “*prescription of the painkillers.”*

In other instances, despite disclosure by the patient, some medical practitioners remained ambivalent to T&CM use and were reluctant to enter a discussion or provide definitive advice (Table 5). Participants accounted for their negative experiences with discussing T&CM by suggesting the medical profession was limited by the lack of scientific evidence available for a T&CM (Table 5). In addition to insufficient evidence, participants also spoke about their frustrations with perceived professional and cultural divides between T&CM and mainstream medicine (Table 5). Throughout, participants called for a patient-centred approach that is respectful of the patient’s views and preferences that considers them a valid member of the decision-making team (Table 5).

### Difficulties with access

The location of available IO services, preferences for service location, and the logistics of accessing them were identified by both interviewees and survey respondents as important barriers (Table 5). Logistics (e.g. no transport) and personal factors (e.g. too unwell or not enough time) accounted for 11% (*n* = 11/84) and 6% (*n* = 9/84) respectively of the reasons for not using any or more T&CM therapies.

Two-thirds of the survey respondents (65%, *n* = 70/107) wanted to access T&CM services across a range of service settings, with 22 (21%) wanting to access IO inpatient and outpatient services; 23 (21%) following inpatient care, either as an outpatient or through their primary care clinic; and 25 (23%) across the continuum of primary and secondary care services including nonmedical community settings. Some participants however, preferred to access T&CM in non-medical settings. Of the 43 (40%) survey respondents who selected a ‘community health centre’ as a preferred site, 11 (10%) only wanted to access these services in non-medical environments as *“Hospitals make you feel ‘you are sick’,”* whereas a community centre *“can mean it’s just ‘your time”.*

Results from the survey however, identified an apparent mismatch between where participants were accessing T&CM services and their preferences. Around two-thirds (69%, *n* = 74/107) of the survey respondents reported that they wanted to access T&CM services at home or close to home, yet only 23% (*n* = 22/95) had accessed T&CM services at home. The desire to access T&CM through the hospital (either as inpatient and/or outpatient) was reported by 64% (*n* = 69/107) of the survey respondents. However, only 17% (*n* = 16/95) had accessed T&CM in a hospital setting. Access via a medical practitioner in the community was also low with 14% (*n* = 13/95) accessing T&CM through a GP clinic, and 26% (*n* = 27/107) selecting this location. A better match was observed between respondents who wanted to access T&CM through a community health centre or private T&CM provider (45%; *n* = 43/95) and those who did (42%; *n* = 45/107).

Participants reported that structural barriers, such as difficulties in accessing T&CM that was distant from their home or not available at the hospital where they were actively being treated, were often compounded by personal barriers such as a lack of energy to seek further treatments and/or the consequences of travel on their health (Table 5). A few participants commented that *“parking”* at the clinical sites that offer IO services was another structural barrier to access, with disability parking providing access for some. Having IO services close to public transport was offered as one solution; so too was community transport. These solutions however, could not completely resolve the challenges of managing personal health constrains and travelling to access such services.

Other personal barriers, such as *“Just too many health appointments”, “Just unable to find the time”*, and logistical issues related to timing, were also reported by participants. The various accounts given by participants demonstrate that cancer survivors experience a variety of practical difficulties that prevent them from accessing IO services.

### Cost of care

Out of pocket costs were a significant personal barrier identified by interviewees and survey participants to accessing IO services that reflected a combination of a lack of funding and affordability (Table 5). In the survey, 82% (*n* = 69/84) selected finance as an important obstacle, from which half reported it was the only obstacle. Participants also emphasised the importance of the continuity of IO services for their effectiveness and, owing to the long recovery time following cancer treatment, the importance of ongoing funding and the need for specialised funding arrangements for IO for cancer patients.

A variety of reasons were given as to why cost was a barrier (Table 5). Participants described the costs of their conventional cancer treatment and the ongoing financial pressures of long-term illness as considerable, leaving little left to pay for extra services such as IO. The limits of private health insurance (high premiums and minimal rebates for T&CM) posed an additional cost barrier to accessing T&CM. Participants complained about the rebate amount received and the limitations placed on the number of sessions they were allowed.

When participants were asked who they thought should pay for all or some of the cost, structural solutions were often suggested. Most of the survey respondents (86%, *n* = 92/107) indicated that the national health service (i.e. Medicare) should pay for these services, followed by 48% (*n* = 52) selecting private health insurance and only 23% (*n* = 25) thought the patient should directly pay for IO services. Participants saw the Medicare scheme as a just reward for a lifetime of work and taxes, maintaining that the government should pay for IO through an appeal to rising social inequality (Table 5).

Failing adequate subsidies from health services and insurers, reliance upon family members was identified as a way to help cover costs. Some patients reported that they had searched overseas to improve affordability of T&CM products. Personal constraints associated with engaging in current cancer treatment made it difficult for many to *“shop around”* for the most affordable services, or to pursue other ways of paying for T&CM such as accessing acupuncture from a medical practitioner.

The attitude that IO services were a luxury item was thought to partly explain why both the public and private health services and insurers were reluctant to fund T&CM. Conversely, participants described T&CM therapies as *“an essential part of recovery”,* and this was justification for more funding to either partially, or completely, subsidise the cost of IO services (Table 5).

## Discussion

The results from the community survey and focus group interviews align with the findings from other Australia studies [3, 11, 30, 31, 37, 38]. A substantial number of cancer survivors use biological based T&CM therapies and consult T&CM practitioners, both during and after active cancer treatment. Participants expressed a clear preference for their oncology team to informed about their T&CM use and the need for greater integration of high quality T&CM services with their other cancer care services. The demand for IO services appeared to far exceed service provision. Out of pocket costs and insufficient information about IO options were significant barriers that exacerbated unmet need and inequalities. The preferred location of IO services, be they collocated with other cancer services in the hospital or in the community close to home, reflected the logistics of accessing care, severity of impairment and disability, and the location of other concurrent cancer care. Owing to the long recovery time following initial cancer treatment, continuity of IO care was also emphasised.

This mixed-method study is one of the first in Australia to specifically explore the views of cancer survivors from CALD backgrounds about IO services. Previous research in Australia has mostly focused on T&CM use rather than IO services [30, 31, 38–42], or the need for supportive cancer care services more generally [43, 44].

The findings from this study also aligned with previous research about the reasons for T&CM use, that is, to augment cancer treatment, increase chances of survival, enhance the immune system, manage side effects, improve quality of life and support self-care [6, 30, 45, 46]. Participants also emphasised that IO addresses their holistic healthcare needs, helps manage comorbidities, and is an essential part of their ongoing supportive care and rehabilitation.

Similarly, among the cancer survivors who did not use, or discontinued using T&CM, the reasons given generally aligned with previous research that identified cost or lack of time; safety concerns, including interactions with conventional cancer care; and a lack of evidence as key reasons [7]. In contrast however, inadequate information about T&CM options and IO services was another important reason for non-use.

Participants expressed the need for greater availability of IO services, more affordable IO services, adequate information about IO management options, and improved coordination of care. Accounts of participants suggest that for the most part, it is the cancer survivors and their caregivers who are left with the responsibility of identifying and integrating T&CM therapies and practices. Information about IO options are often limited, particularly for CALD groups. Often, discussions about T&CM with the patient’s oncology team occurs on an ad-hoc basis and is constrained by the healthcare professionals’ attitudes and knowledge.

The most substantial barrier to accessing T&CM or more T&CM however, was affordability. The substantial financial burden and out-of-pocket costs to cancer survivors in Australia is well documented, affecting patients in both the public and private healthcare sectors [23, 47]. Participants acknowledged the various challenges with funding IO services and suggested higher rebates from private health insurers and more public funding for the underserved would improve affordability, access and equity. These opinions were in contrast to the findings from a comparable Australian study where the focus group participants considered self-funding to be acceptable and did not expect T&CM to be publicly funded [11]. The generalisability of the earlier study however, was limited by a small sample of patients from a single oncology unit. The challenges with funding and under provision of services are not unique to IO and reflect the increasing unmet needs for all types of supportive cancer care services [44, 48].

The qualitative methods used for the focus group interview enabled the research team to engage with, and explore the views of, CALD groups and this was achieved with varying levels of success through the representation of cancer survivors and caregivers of Chinese, Vietnamese and Arabic backgrounds. In navigating a foreign healthcare service, their additional challenges reflect general cultural, language and information barriers that are not necessarily unique to IO [26].

The widespread integration of T&CM in Chinese cancer centres in East Asia, particularly in mainland China, has enabled the building of clinical expertise in IO [49]. It should not be surprising then that many participants in the Chinese and Vietnamese focus groups expressed the need to access similar IO services in Australia. In contrast, the Arabic participants had not used T&CM as part of their cancer care and although they were interested in such services, were unaware that IO options existed. The reason is not clear. The observation might be a sampling artefact, as only 11 like-minded people in one focus group were interviewed. Although the interviewers specifically inquired about other customary and traditional healing practices, there may still have been communication or cultural barriers since there is evidence, that traditional Islamic and Arabic plants are used for cancer care and general health [50]. Conversely, there is also evidence suggesting that many Arabic-Australians have uncritically embraced the Western medical model, with community leaders expressing concerns about an over reliance on multiple prescription medications [51]. Questions about that prayer were not asked, as pastoral care were outside the scope of this research project. This may partly explain the finding that no one had used T&CM as population surveys consistently observe that prayer and spiritual practices are one of the most commonly used non-biological based T&CM therapies in Australia and Arabic countries alike [3, 30, 31, 52, 53].

Limitations of this study include missing data about the participants’ gender and the sampling strategies that lead to an underrepresentation of CALD groups in the on-line survey, of non-T&CM users (and conversely for T&CM users from Arabic-Australian backgrounds) in the focus group interviews, and other common CALD groups in Australia, including Aboriginal and Torres Strait Islander peoples in both the on-line survey and focus group interviews. As such, data saturation may not have been achieved, which in turn limits the generalisability of the findings. Different methodological approaches and recruitment strategies should therefore be employed to obtain more accounts about demands and barriers to integrating T&CM with conventional care and the provision of IO services.

Results from the quantitative survey data must also be interpreted with caution. Statistics about participants’ characteristics, the use of T&CM, and estimated unmet needs should not be extrapolated and only used to provide contextual background information about the respondents. The sample from the community survey was skewed to women who had a diagnosis of breast cancer and users of oncology massage. This reflected the social media networks used to recruit participants to the community survey. As such, the survey sampled a significantly higher percentage of T&CM users than what is thought to be the rate in Australia [30]. However, even the T&CM users experienced substantial barriers to engagement with T&CM services and Australian cancers survivors with a diagnosis of breast cancer have been found to have the lowest levels of unmet supportive care needs [44]. Therefore, if anything, the survey was likely to have underestimated rather than overestimated unmet needs.

## Conclusions

Cancer survivors are high users of T&CM and are using these therapies before, during and following active cancer treatment [3, 11, 30, 31, 37–42]. Given the demand for T&CM services and a preference by some cancer survivors for these services to be integrated with their conventional cancer care, it is important that oncology teams are well informed about the benefits, risks and indications for T&CM use and consider how these services might be provided. This study provided preliminary data to suggest that supportive care services that includes IO, are an important component of quality care in Australia and should not be automatically relegated to a luxury ad-on service for those who can afford them. The challenges with funding IO services and the high out-of-pocket costs for patients were significant barriers that exacerbated inequitable access, particularly for Australians in lower socioeconomic groups, including cancer survivors from some CALD groups. Ongoing consultation with cancer survivors is key to appropriate service provision. The health behaviours, preferences and unmet cancer care needs of cancer survivors from a diverse range of CALD and Indigenous backgrounds warrants further in-depth attention.

## Additional files

Additional file 1: Focus group questionnaire and interview guide. Outline of interview schedule. (PDF 40 kb)

Additional file 2: Online survey questionnaire. Survey questionnaire. (PDF 83 kb)

## Abbreviations

CALD: Culturally and linguistically diverse
FG: Focus group
GP: General practitioner
IO: Integrative oncology
T&CM: Traditional and complementary medicine
