# Supplementary material for: Australian integrative oncology services: a mixed-method study exploring the views of cancer survivors
Source: BMC Complement Altern Med. 2018 May 9;18:153. doi: 10.1186/s12906-018-2209-6 (PMC5944107; doi:10.1186/s12906-018-2209-6)
Supplement: Supplementary file 1 — Focus group questionnaire and interview guide. Outline of interview schedule. (PDF 40 kb) [file 12906_2018_2209_MOESM1_ESM.pdf]

Australian integrative oncology services: a mixed-method study exploring the views of cancer survivors (Hunter, J. Ussher, J. Parton, C. Kellett, A. Smith, C. Delaney, G. Oyston, E.)

Additional File 1: Focus group questionnaire and interview guide

Focus Group Participant Details

1. What is your current age? (in years)
2. What is your current postcode?
3. What it your country of birth?
4. With which ethnic or cultural group do you identify?
   (e.g. Anglo-Australian, Chinese)
5. Which language do you speak at home?
6. What type of cancer were you diagnosed with?
7. What stage of treatment are you in currently? (Please circle)
   Active treatment / Long-term treatment / No treatment
8. How long ago were you diagnosed with cancer?
   Years: Months:

Interview Guide

T&CM use:

1. What kinds of CM have you heard about?
2. What CM have you used? Which ones, why?
3. Regarding your cancer - did you use CM in addition to conventional medical care or instead of conventional treatment? Which ones, why?
4. Have you used CM for other reasons now or in the past?
5. Have you benefited from using CM or know other people who have benefited?
6. Would you consider starting/continuing to use CMs in the future? Which ones, why?

CM decision making:

1. What influences your decision to use CM?
2. Has anyone recommended you use CM? Who, which ones, why? Did you accept their advice?
3. If a doctor recommended CM, would you use it?
4. If a nurse or other health care practitioner recommended CM, would you use it?
5. What about friends or family members?
6. What about community leaders?

CM delivery:

1. What are your thoughts about coordinating CMs with conventional care?

2. What role would you want and/or expect the following members of your treating team to have in terms of CMs? Oncologist, GP, nurse, psychologist, physio etc
3. Would you expect them to refer you, recommend it, provide information, talk about CM?
4. Where would you like to access CM? Whilst an inpatient, attending outpatient department, at the GP clinic, in a community centre, close to home?
5. Where would you prefer CM services to be provided?

CM funding:

1. How do you pay for CM?
2. Who should pay for CM?
3. Do you think it should be an “extra” that you pay for, that private health insurance should pay for, or funded through the public health system?

CM information:

1. Where do you get information about CM?
2. What information sources do you trust?
3. What further information do you need?
4. What are the best formats – verbal, written (which language), internet

CM barriers:

Is anything stopping you from using CM or using more CM? (prompts if needed)

1. Availability?
2. Financial?
3. Logistical? e.g. too far, no transport
4. Personal? e.g. too sick, not enough energy or motivation
5. Health practitioners unsupportive?
6. Family or friends unsupportive?

©2017 NICM, Western Sydney University, Australia
