# Supplementary material for: Australian integrative oncology services: a mixed-method study exploring the views of cancer survivors
Source: BMC Complement Altern Med. 2018 May 9;18:153. doi: 10.1186/s12906-018-2209-6 (PMC5944107; doi:10.1186/s12906-018-2209-6)
Supplement: Supplementary file 2 — Online survey questionnaire. Survey questionnaire. (PDF 83 kb) [file 12906_2018_2209_MOESM2_ESM.pdf]

Australian integrative oncology services: a mixed-method study exploring the views of cancer survivors (Hunter, J. Ussher, J. Parton, C. Kellett, A. Smith, C. Delaney, G. Oyston, E.)

Additional File 2: Online survey questionnaire

Instructions

Taking part in the study is voluntary. Information you provide is confidential.

The survey will take about 15-20 minutes to complete.

Please do not exit the survey before completing as you cannot return to the incomplete parts after exiting.

Use the previous and next buttons at the bottom of each page to move between pages. If you have any questions, please contact us on: Tel: 02 4620 XXXX; Email: XXXXXX

Your participation in this study is greatly appreciated.

These questions are about you and your experience of cancer

1. What is your current age?

2. What is your current postcode?

3. What is your country of birth?

4. With which ethnic or cultural group do you identify? (e.g. Anglo-Australian, Chinese)

5. What language do you speak at home?

6. What type of cancer were you diagnosed with?

7. What stage of treatment are you in currently?
    a) Active treatment
    b) Long-term treatment
    c) No treatment
    d) Other (please specify)

8. How long ago were you diagnosed with cancer? Years/Months

9. Which of the following have you used/had in the last 12 months for your cancer treatment?
   a) Radiotherapy
   b) Chemotherapy
   c) Surgery
   d) Other cancer treatment medicines (e.g. tamoxifen)
   e) None

These questions are about your experience of complementary therapies since your diagnosis with cancer

10. Please select any of the following therapies that you have used either during your cancer treatment or to help with your recovery?
    a) Oncology massage
    b) Other massage therapy
    c) Reflexology
    d) Aromatherapy
    e) Therapeutic touch / Reiki
    f) Chiropractic
    g) Osteopathy
    h) Cranio-sacral
    i) Yoga
    j) Tai Chi
    k) Qi Gong
    l) Relaxation
    m) Meditation
    n) Music therapy
    o) Acupuncture
    p) Chinese medicine
    q) Ayurvedic medicine

©2017 NICM, Western Sydney University, Australia

r) Naturopathy

s) Indigenous Australian Medicine

t) Nutritional medicine

u) Other (please explain)

11. Please indicate whether you selected any of the therapies in the previous question.

If YES go to Q13; if NO go to Q12

12. Have you used any vitamins, minerals, herbs, fish oil, probiotics, or other natural health products either during your cancer treatment or to help with your cancer recovery? Y/N

If YES go to Q18; if NO go to Q22

13. Have you used any vitamins, minerals, herbs, fish oil, probiotics, or other natural health products either during your cancer treatment or to help with your cancer recovery? Y/N

14. Who recommended you use these complementary therapies or natural health products? Please select all that apply.

a) A doctor from the hospital or oncology service

b) A nurse or another practitioner working in the hospital

c) A General Practitioner (GP)

d) A pharmacist

e) A natural health practitioner

f) Friend or family member

g) I decided to use them

h) Other (please explain)

15. Where did you access these complementary therapies or natural health products?

a) The hospital or oncology clinic

b) General practice clinic or medical centre

c) Health clinic

d) In a community centre or hall

e) At a friend's house

f) In my house

g) Other (please explain)

©2017 NICM, Western Sydney University, Australia

16. In what way did you use natural health products?

17. Would you recommend any of these complementary therapies or natural health products to a friend in a similar situation? Please explain why.

Go to Q23

18. Who recommended you use these natural health products? Please select all that apply.
    a) A doctor from the hospital or oncology service
    b) A nurse or another practitioner working in the hospital
    c) A General Practitioner (GP)
    d) A pharmacist
    e) A natural health practitioner
    f) Friend or family member
    g) I decided to use them
    h) Other (please explain)

19. Where did you access these natural health products?
    a) The hospital or oncology clinic
    b) General practice clinic or medical centre
    c) Health clinic
    d) In a community centre or hall
    e) At a friend's house
    f) In my house
    g) Other (please explain)

20. In what way did you use these natural health products?

21. Would you recommend any of these natural health products to a friend in a similar situation? Please explain why.

Go to Q23

22. If you haven't used any of the complementary therapies or natural health products listed previously, why not?

23. Are there complementary therapies you wanted to use but couldn't? If so, why not?

24. Would you consider using complementary therapies in the future? Which ones and why/why not?

25. Do you think oncology services should offer complementary therapies? Y/N
If so, what services and where?

26. Where would you like to access complementary therapies?

    a) Whilst an in-patient

    b) Attending an out-patient department

    c) GP clinic

    d) Community centre

    e) Close to home

    f) Other (please specify below)

    Please explain your preferences.

27. How would you like to be referred to a complementary therapy service?

    a) Through an oncologist or GP

    b) Through self-referral

    c) Other (please explain)

28. Would you want your oncology team to know you are using complementary therapies?

29. Who do you think should pay for complementary therapies?

    a) Medicare

    b) Private health insurance

    c) Person funds

    Please explain your preferences

©2017 NICM, Western Sydney University, Australia

30. What is stopping you from using complementary therapies or using more complementary therapies?

    a) Availability

    b) Finance

    c) Logistics e.g. no transport

    d) Person e.g. too sick

    e) Health practitioner / doctor unsupportive

    f) Family or friends unsupportive

    Please explain

THANK YOU FOR COMPLETING THIS SURVEY

©2017 NICM, Western Sydney University, Australia
